# Exploring the Impact of Cyanidin-3-Glucoside on Inflammatory Bowel Diseases: Investigating New Mechanisms for Emerging Interventions

**DOI:** 10.3390/ijms24119399

**Published:** 2023-05-28

**Authors:** Maximos Frountzas, Eva Karanikki, Orsalia Toutouza, Demosthenis Sotirakis, Dimitrios Schizas, Panagiotis Theofilis, Dimitris Tousoulis, Konstantinos G. Toutouzas

**Affiliations:** 1 First Propaedeutic Department of Surgery, Hippocration General Hospital, School of Medicine, National and Kapodistrian University of Athens, 11527 Athens, Greece; 2 Department of Clinical Nutrition, Hippocration General Hospital, 11527 Athens, Greece; karanikki.eva@gmail.com; 3 School of Medicine, Imperial College of London, London SW7 2AZ, UK; 4 First Department of Surgery, Laikon General Hospital, School of Medicine, National and Kapodistrian University of Athens, 11527 Athens, Greece; 5 First Cardiology Department, “Hippocration” General Hospital, University of Athens Medical School, 11527 Athens, Greece

**Keywords:** C3G, anthocyanin, IBD, ulcerative colitis, Crohn’s disease

## Abstract

Cyanidin-3-O-glucoside (C3G), the most widely distributed anthocyanin (ACN) in edible fruits, has been proposed for several bioactivities, including anti-inflammatory, neuro-protective, antimicrobial, anti-viral, anti-thrombotic and epigenetic actions. However, habitual intake of ACNs and C3G may vary widely among populations, regions, and seasons, among individuals with different education and financial status. The main point of C3G absorption occurs in the small and large bowel. Therefore, it has been supposed that the treating properties of C3G might affect inflammatory bowel diseases (IBD), such as ulcerative colitis (UC) and Crohn’s disease (CD). IBDs develop through complex inflammatory pathways and sometimes may be resistant to conventional treatment strategies. C3G presents antioxidative, anti-inflammatory, cytoprotective, and antimicrobial effects useful for IBD management. In particular, different studies have demonstrated that C3G inhibits NF-κB pathway activation. In addition, C3G activates the Nrf2 pathway. On the other hand, it modulates the expression of antioxidant enzymes and cytoprotective proteins, such as NAD(P)H, superoxide dismutase, heme-oxygenase (HO-1), thioredoxin, quinone reductase-oxide 1 (NQO1), catalase, glutathione S-transferase and glutathione peroxidase. Interferon I and II pathways are downregulated by C3G inhibiting interferon-mediating inflammatory cascades. Moreover, C3G reduces reactive species and pro-inflammatory cytokines, such as C reactive protein, interferon-γ, tumor necrosis factor-α, interleukin (IL)-5, IL-9, IL-10, IL-12p70, and IL-17A in UC and CD patients. Finally, C3G modulates gut microbiota by inducing an increase in beneficial gut bacteria and increasing microbial abundances, thus mitigating dysbiosis. Thus, C3G presents activities that may have potential therapeutic and protective actions against IBD. Still, in the future, clinical trials should be designed to investigate the bioavailability of C3G in IBD patients and the proper therapeutic doses through different sources, aiming to the standardization of the exact clinical outcome and efficacy of C3G.

## 1. Introduction

Over the last years, scientific data support that many non-communicable chronic diseases (NCCDs) could be prevented through sufficient dietary intake of bioactive molecules derived from fruits and vegetables. Soluble and insoluble dietary fibers, antioxidants, functional carbohydrates and polyunsaturated fatty acids are responsible for several health benefits. More specifically, dietary antioxidants (AOX), such as pro-vitamins and phenolic compounds (PC), including anthocyanins (ACNs; anthos = flower, kianos = blue), could alleviate the oxidative stress associated with different molecular events in the body [1]. ACNs are responsible for several bioactivities, including anti-inflammatory, neuro-protective, antimicrobial, anti-viral, anti-thrombotic, and epigenetic actions [2]. However, ACNs differ in terms of metabolic fate and bioactivity. Several physiological barriers in the human body as well as various physical and chemical components in natural or prepared plant foods could influence their metabolic action [3]. In fact, the structure of ACNs is responsible for their nutraceutical potential, which has been associated with their specific physicochemical behavior within foods and biological systems [4]. Similarly, lots of data about Cyanidin-3-O-glucoside (C3G), the most widely distributed anthocyanin in edible fruits, have been published over the previous ten years and will be discussed in the following paragraphs [5]. 

Inflammatory bowel disease (IBD), which mainly presents as Crohn’s disease (CD) or ulcerative colitis (UC), is a heterogenous chronic bowel inflammation without clear etiologic factors. Typically, the first manifestation is a complicated immune response that comprises a number of inflammatory cells, including macrophages, monocytes, neutrophils, natural killer cells, cytokines, and chemokines [6]. The subacute course of this clinical entity is further characterized by chronic immunological reactions that aim to balance local provocative factors and healing processes [7]. Alterations in gut microbiota seem to play a role in maintaining the above-mentioned immunological balance. Several therapeutic agents, such as corticosteroids, immunomodulatory drugs or targeted therapies, have been suggested through years, but there is still lack of clear evidence about the efficacy of several therapeutic regimens [8].

In recent years, the need for more effective therapeutic strategies against IBD has emerged. Under these circumstances, several biological agents with anti-inflammatory or antioxidative functions have been investigated in terms of their safety and efficacy against IBD [9]. The aim of this review is to investigate the possible therapeutic properties of Cyanidin-3-O-glucoside (C3G) in IBD.

## 2. Cyanidin-3-O-Glucoside

### 2.1. Chemical Structure

ACNs are anthocyanidin glycosides. Their backbone consists of a benzopyran core [benzoyl ring (A), pyran ring (C)], a phenolic ring (ring B) attached to its 2-position, and a sugar moiety, mainly, at its 3-position in the C-ring. Some 31 anthocyanidins (aglycones) and more than 600 ACNs have been determined thus far [10]. However, the majority of ACNs are based on six aglycones that differ in their B-ring substitution pattern: cyanidin (Cy), delphinidin (Dp), pelargodin (Pg), peonidin (Pn), petunidin (Pt) and malvidin (Mv). These aglycons are further divided into groups based on the type and amount of bonded sugars, as well as the presence of aliphatic or aromatic carboxylates (attached to their sugar moieties). The three non-methylated aglycones (Cy, Dp, and Pg) are further classified as 3-monosides (mostly glucosides), 3-biosides, 3,5- and 3,7-diglucosides [11]. Moreover, the heterogeneity in ACNs’ structure represents a challenge for their isolation and identification. However, according to Flamini et al., the mass spectral fingerprint of Cy and the majority of its glycosides have been established so far [12].

The processes of glycosylation and methylation affect Cy’s hydrophobic (octanol)/hydrophilic (water) partition coefficient (LogP), its polar surface area (Å^2^), and its molecular weight (MW). All of these have important implications in the metabolic fate of Cy-derivates (ADME: absorption, distribution, metabolism and excretion). Cy has a lower MW (287.24 g/mol), and Å^2^ (114.3) is less hydrophilic (LogP = 3.05) than C3G (449.4 g/mol, Å^2^ = 191, LogP = 0.39). A second glycosylation (Cy-3,5-O-diglucoside, Cy3,5GG) increases its hydrophilic character, but compromises its absorption capacity, while an extra malonyl group (Cy3MG) has the opposite effect [13]. In addition, other structural features in C3G have important implications for its chemical reactivity in vitro. STD-NMR spectroscopy and molecular dynamics simulations have been utilized to show that the absence of an extra hydroxyl at R^5′^ in C3G affects its binding capacity toward citrus pectins, when compared to Dp3G [14]. Moreover, it has been demonstrated that C3G (as a flavylium cation, pH 3.4) binds spontaneously within 1 min to bacterial (*Gluconacetobacter xylinus* ATCC 53524)-derived cellulose [15]. It has also been reported that this binding behavior is not limited by the available interacting sites in cellulose, but it is limited by the number of free C3G molecules. Therefore, a Langmuir binding isotherm model is proposed:

*Q* = *Q_max_* × [(*K_L_·C*) × (1 + *K_L_* × *C*)^−1^].

where *Q* represents the amount of absorbed C3G per unit mass of cellulose (µg·mg^−1^), *Q_max_* is the apparent max adsorption capacity (1109 µg·mg^−1^ of cellulose), *K*_L_ is the apparent binding affinity constant, and *C* is the free C3G concentration at equilibrium (mM). By applying this equation, a “C3G saturation effect” could be observed at about 200 mM. Furthermore, utilizing an in vitro model to simulate GI conditions demonstrated a “bind–release” behavior between C3G and pectin/chitosan at each digestion step (oral, gastric, and intestinal). On the other hand, it is suggested that this polymeric mixture functions as a protective mechanism against G3G degradation, as it is gradually released from protein and polysaccharide bonds and eventually becomes available for absorption by GI epithelial cells [16].

It is highly important that C3G binds to proteins in vitro as well. ACNs present different binding capacities to human serum albumin (HSA; Dp3G > C3G > Pg3G), but their capability to induce structural changes to this protein is also different (Pg3G > C3G > Dp3G) [17]. In addition, C3G–protein interactions are established by hydrogen bonding and van der Waals forces; thus, the secondary structure of bovine serum albumin (BSA), hemoglobin (Hb), and myoglobin (Mb) are almost destroyed (less% α-helixes) [18]. C3G is usually represented as a cation, which is only possible under acidic conditions, such as those created by gastric juice, and in silico assays, which have revealed that cationic C3G cannot be absorbed through passive diffusion. Nevertheless, only a substitution at R3^1^ [–H (Pg3G) by –OH (C3G)] modifies C3G’s bioaccessibility, absorptivity, and metabolism within enterocytes [19].

Finally, rare types of anthocyanidins, such as 3-deoxy-anthocyanidins, hydroxylated at the 6th position, 5, 7, 3^1^, 5^1^-*O*-glycosilated, *C*-glycosylated, or aliphatic (mainly malonic and pyruvic acids)- and PC-acylated ACNs are also currently studied, as they seem to be more bioactive than their conventional counterparts [20]. For example, Cy-malonyl-glucoside (Cy-Mal-3G) demonstrates stronger anticancer (colon, liver, prostate, and breast) activity than C3G [21]. Moreover, C3G acylated with lauric acid improves its stability due to its ester group, which is more stable than a hydroxyl group [22]. 

### 2.2. Dietary Sources

C3G is one of the commonest but not the major cyanidin; black elderberry, blue hybrid maize and Korean black raspberry are exceptions to this rule [23]. The daily intake and further bioactivity of C3G are highly affected by the proper selection of their plant sources. Some classic fruits characterized by increased C3G bioavailability are pomegranate and blackberry, while some exotic fruits are bilberry, elderberry and mulberry [24]. Although C3G is widely known to be found in fruits, mainly in berries and other blue and red fruits and vegetables, it is not necessarily the main ACN. For example, strawberry has 15 times more Pg3G, and raspberry (fresh/pomace) has 1.4–1.5 times more Cy-3-*O*-sophoriside (Cy3So) than C3G [25].

Dietary surveys with detailed information on the total and specific intake of ACNs are also scarce. Daily intake of ACNs, mainly C3G, does not depend on the richness of their sources. Mean daily intake per capita of ACNs was approximately 12.5 mg in US adults in 2000–2002, 80% coming from blueberry, grape, onion, grape 100% juices, raspberry, red cabbage, wine, and cherry sweet. In 2007–2008, the mean daily intake was 11.2 mg, adding red/purple vegetables, bananas, and yogurt to the list [26]. In Europeans, the mean intake of total ACNs is about 20 mg/d, with Cy being the most common, and in Polish adults participants of the HAPPIEE study, 56% of daily ACN intake came from blackcurrants, beans, and strawberries [27]. Eastern countries present a higher intake of flavonoids than Americans or Europeans, but their ACN sources appear to be lesser than their isoflavone/proantocyanidin sources [28]. According to KNHANES 2007–2012, the mean daily intake of flavonoids was 318 mg/d/person. Some 23% of this mean daily intake was from proanthocyanidins, and 11.6% from anthocyanidins, while 20.3% was from flavonones, and 0.3% from flavones. The major contributing food groups to flavonoid intake were vegetables (20.5%) such as onions (9.6%) and fruits (54.4%) such as apples (21.9%), mandarins (12.5%), grapes (9.0%), and other fruits (1.4%) [29]. It should be noted that habitual intake of ACNs and C3G may vary widely among populations, regions, and seasons, and among individuals with different education and financial status, and depends on adequate dietary assessment tools (e.g., 24FR vs. FFQ) [30]. 

In conclusion, dietary choices could have an important impact on both ACN (and C3G) intake and the following health effects. In this sense, recent progress in agricultural and food technology has driven the international market of berry fruits at a lower cost [31]. According to the report of the Agri-Food and Fisheries Service, the production of berries in Mexico has increased almost three-fold in recent years [32]. Therefore, intake of ACNs and particularly of C3G is expected to gradually increase over the next years. 

### 2.3. Bowel Metabolism

The small bowel is the third location of C3G metabolism, after the oral cavity and stomach. Unlike gastric conditions, the physical and chemical microenvironment in the small intestine reduces C3G’s bioavailability by 40–50% [33]. Factors such as pH, C3G’s ability to release from the food matrix, pancreatic and brush border enzyme action, transportation processes and enterocyte metabolism in phase I/II are crucial for the bioavailability of C3G, Cy and their metabolites (degradation products or phase II metabolites). At intestinal pH (8.2 ± 0.2), C3G becomes negatively changed and highly unstable, returning to its quinoidal form, while its glucose moiety remains neutral. Moreover, further de-glucosylation of C3G is performed with neither lactase-phlorizin hydrolase (LPH; EC 3.2.1.62) nor cytosolic β-glucosidases [34]. It is worth mentioning that the cleavage of its glucose moiety is not a prerequisite for C3G chemical breakdown and splanchnic metabolism, although it is an important mediator of its trans-epithelial transport [35].

Once inside the enterocyte, Cy and C3G could be either transformed to other PC (particular phenolic acids) and derivatives in phase I metabolism or to several conjugates (methylated, glucuronidated or sulphated) in phase II metabolism [36]. Microbials, with the exception of metabolic machinery, are responsible for producing phase I metabolites, although there is controversy as to whether this process occurs in the small intestine or as a product of the enterohepatic cycle (EHC) [37]. In phase II metabolism, many enzymes, such as phenyl sulfotransferases (PST), uridine 51diphosphate glucuronosyltransferases (UGT) and catechol-O-methyltransferase (COMT), may modify the Cy (and other anthocyanidins) structure, making it more water-soluble and thus facilitating their further elimination by the kidneys [13].

The main derivatives of Cy’s metabolism/degradation are protocatechuic acid (PCA) and phloroglucinaldehyde (PGA) [38]. Cy and PGA, due to their hydrophobic nature in comparison to PCA, can passively diffuse through biological membranes, reaching the plasma in the first 2 h. Another reported reaction involves Cy (or C3G) methylation to produce Pn (or Pn3G), with both having almost the same in vivo bioactivity [39]. Recently, the major pathways for C3G metabolism in liver microsomes have been proposed. After deglycosylation, Cy produces PGA from its A-ring, ferulic acid (FA), 3,4-dihydroxyphenyl acetic and 4-hydroxyphenylacetic acids from its B-ring, and 3,4-dihydroxybenzaldehyde (PCA immediate precursor) also from its B-ring [40].

C3G and derived metabolites that surpassed absorption from the small bowel can finally be released from fibrous food matrices (also known as macromolecular antioxidants), transformed by the microbiome, and then absorbed by colonocytes [41]. The large bowel contributes to the remaining deglycosylation, phenolic acid production, and phase II conjugation events, resulting in the excretion of, intact, less than 0.005% of C3G. As occurred in the small bowel, the C-ring rupture and the Cy chalcone formation lead to the apparition of hydroxybenzoic’s (OH-BA) and phenylacetic acids’ derivatives, VA, IVA, FA, and HA, which can further be eliminated in feces and urine (by means of EHC) [42]. These metabolic transformations are mediated by the slightly basic pH present at this level, where C3G and Cy are highly unstable. On the other hand, certain metabolites such as 2-OH-4-methoxybenzoic acid, 4-methoxybenzaldehyde, methyl-VA, and caffeic acid are specifically produced within the large bowel [43].

### 2.4. Health Effects

C3G’s bioactivity has been further investigated in clinical studies in humans. For instance, C3G benefits cardiovascular health [44,45,46]. A double-blind, randomized crossover study indicated that some ACNs peaked within 1–3 h, just as HDL-cholesterol did [placebo vs. 640 mg/d/4 weeks (purified ACNs supplement)] [47]. However, when different ACN sources were utilized in the same protocol [placebo vs. 486 mg/d/4 weeks (Delphinol^®^ from maqui berry, Huechuruba, Santiago, Chile], a decrement in LDLox and F2-isoprostanes levels was highlighted [48]. However, both studies did not report any other noticeable benefit for CVD markers [49]. According to this, the significant changes that were reported were upregulation in serum HDL and downregulation in LDL cholesterol; in addition, soluble vascular cell adhesion molecule-1 (sVCAM-1) and high sensitivity C-reactive protein (hsCRP) levels were observed after 24-week ACNs = supplementation [50]. The same results have been reported with ACNs from strawberries, which modulated these inflammatory biomarkers and indirectly improved insulin action [51]. It is noteworthy that endothelial health is correlated to the regulation of nitric oxide production, and the latter could be altered by several types of flavonoids, including ACNs [52]. As for C3G’s anticancer activity, Cy derivatives (including C3G) from black raspberries (BRB) are extensively metabolized and retained within the oral cavity of healthy humans. In addition, administrating per os troches of freeze-dried BRB to oral squamous cell carcinomas patients (OSCCs) for 14 days improves the expression of pro-survival genes (AURKA, BIRC5, EGFR). It reduces other pro-inflammatory genes (NFKB1, PTGS2) [53]. Moreover, the acute intake of a blueberry dry extract regulates DNA methylation in patients with colorectal adenocarcinomas, despite the inter-individual variability [54].

The radical scavenging capacity (RSC) and molecular competition ability of C3G (and Cy) may help prevent certain inflammatory processes, CVD, aging, and cancer [55]. For instance, senescent and cancer cells are also susceptible to DNA cleavage due to epigenetic factors that induce the production of free radicals and activate oxidative enzymes, such as xanthine oxidase, which can be attenuated by Cy and C3G [56]. Additionally, some animal studies have suggested that C3G may slow or inhibit the absorption of carbohydrates (glucose) and lipids in the intestine, confirming the postulated mechanisms in cells and the physiological impact on humans [57]. It has been observed that C3G provides protection against CVD related to oxidative stress, due to its transportation in EA.hy926 cells via a specific bilitranslocase, and it accumulates in the vascular endothelium wherein it exerts anti-ischemic properties on isolated rat heart [58]. It is worth mentioning that C3G’s metabolic bioactivity in adipose cells has been extensively studied, both in vivo and in vitro. C3G and Cy both upregulate human adiponectin, uncoupling acylCoA oxidase-1, protein-2, and perilipin, while they downregulate IL-6 and plasminogen activator inhibitor-1 [59]. When adipose cells are exposed to the omega-3-fatty acid docosahexaenoic acid, C3G suppresses the secretion of interleukin-6 and monocyte chemoattractant protein-1 (MCP-1/CCL2) and decreases its basal lipolytic activity [60]. However, C3G and Cy upregulate the hormone-sensitive lipase gene and enhance the lipolytic activity of rat adipocytes [61]. 

On the other hand, the biological effects demonstrated in laboratory animals and in vitro assays do not precisely reflect their efficacy in humans. In a great number of cases, the amount required to achieve a specific biological action in general is much larger than that obtained from dietary sources. For example, the average amount of C3G employed in rat/mice bioassays far exceeds (by around 30–60 times) the amount that can be obtained from a single dietary source. On the other hand, in different cases, the amount derived from a habitual diet is sufficient to achieve certain benefits [62]. Moreover, C3G from natural sources or manufactured nutraceuticals is seriously restricted by its splanchnic metabolism, and thus its efficacy in targeting internal tissues is limited. Taking C3G’s low bioaccessibility and bioavailability into account, entrapping agents such as malto/cyclodextrins or liposomes may be efficient alternatives to preserve its properties within the GI tract. Under these circumstances, concentrated sources of C3G, such as purees or freeze-dried fruits, provide a much higher intake of C3G, but also preserve its bioactive ability within the GI tract [63]. 

The previously stated bioactivities of C3G, its aglycone (Cy), and derived metabolites mostly rely on the following mechanisms: RSC, epigenetic action, competitive protein-binding, and enzyme inhibition. Flavonoids seem to exert regulatory effects on gene expression. Some examples are naringenin, kaempferol, and quercetin. The combination of flavonoids and chemotherapy seems to be an interesting approach to cancer treatment too [64]. However, molecular studies involving C3G or Cy as epigenetic effectors are still scarce. Many in vivo and in silico studies using pure DNA or protein systems have indicated their macromolecular-binding and enzyme inhibition capacities [65]. A ∆λ to the ultraviolet-visible spectra of this nucleic acid has been demonstrated while studying the binding capacity of Cy and C3G to calf thymus DNA, indicating the formation of the DNA-Cy and DNA-C3G complexes with an intercalative binding mode evidenced in their fluorescence spectra, and with C3G binding to DNA more efficiently than Cy [66]. 

Both molecules also bind salivary and blood proteins, which might modify their fate within GI and their bloodstream transport. Spectroscopic studies suggest that C3G spontaneously binds albumins by means of weak forces such as hydrogen bonds and Van der Waals forces, as well as hydrophobic interaction on a minor scale [17]. C3G binds to BSA to its IIA sub domain and is surrounded by key hydrophobic and non-polar (Ala, Leu, Tyr Phe, Trp and Gly) and polar (Arg, Glu, Lys and Asp) residues within the hydrophobic cavity of site II’ [67]. Similarly, the abovementioned phenomenon has been reported in HAS. Certain structural features of both anthocyanidins/ACNs modify their binding capacity toward HAS; at increased pH 7.0 and at reduced pH 4.0, there is a differential electrostatic environment resulting in differences regarding the binding capacity of ACNs in their quinoidal form [68]. In addition, the binding constant of C3G has been reported to be higher for myoglobin than for BSA hemoglobin, which is structurally associated with its binding capacity toward α-helices. Finally, evidence on the differential binding and inhibitory capacity of Cy and C3G toward GI enzymes has been elucidated. C3G is a much stronger inhibitor of both intestinal α-glucosidase and pancreatic α-amylase as compared to Cy [69], whereas glucose substitution at the 3-O (increases) and 5-O (reduces) positions in Cy modifies its inhibitory activity toward α-glucosidase.

## 3. Inflammatory Bowel Disease (IBD)

Inflammatory bowel disease (IBD) is a term that describes disorders involving long-standing (chronic) inflammation of tissues within the GI tract. Types of IBD include ulcerative colitis (UC) and Crohn’s disease (CD). As far as ulcerative colitis is concerned, it involves inflammation and sores (ulcers) along the lining of large intestine (colon) and rectum [70,71,72]. On the other hand, Crohn’s disease is characterized by inflammation of the lining of digestive tract, which often may involve its deeper layers. Despite the fact that Crohn’s disease most commonly affects the small intestine, it can also affect the large intestine, and, rarely, the GI tract. Both are typically characterized by abdominal pain, diarrhea, rectal bleeding, fatigue and weight loss. Moreover, they are associated with extraintestinal manifestations, such as anemia, fever, weight loss, arthritis, ankylosing spondylitis, sclerosing cholangitis, uveitis, iritis, pyoderma gangrenosum and erythema nodosum. IBD patients also have a higher risk of colon cancer [71]. The extent of the symptoms is not the same for every patient, as it may range from mild illness to life-threatening complications.

### 3.1. Epidemiology

The number of children living with IBD is growing rapidly; in Canada, the diagnosis of young people increased 50% in the first decade of 21st century [73]. In 2018, there were over 7000 children and youth under 18 years old living with IBD in Canada alone, and 600 to 650 young children (under 16 years) diagnosed every year [73]. Several reports have been drafted on the prevalence of IBD in developed Western countries. Asia has a lower prevalence of IBD, whereas a recent increase in its incidence and prevalence has been observed in Eastern Europe and Asia [74]. Previous studies have demonstrated that the prevalence of Crohn’s disease (CD) is higher than that of ulcerative colitis (UC) in pediatric patients in Northern California. However, in French pediatric patients, the observed rate for UC was higher than that for CD [75]. In a survey in Western Europe, IBD was found to affect 0.5–1% of the population, with 56 and 104 new cases per million inhabitants per year for CD and UC, respectively [76]. In addition, IBD affects approximately 1.4 million patients in the USA and 2.4 million in Europe [77]. In another investigation, the incidence of UC was reported to be around 10–20 per 1,000,000 per year, with a prevalence of 100–200 per 1,000,000 in Western countries [78].

### 3.2. Pathogenesis

The etiology of IBD is still not completely understood. Several studies support the hypothesis that its onset originates from the combination and interplay of immune dysregulation (chronic or relapsing), genetic factors, environmental triggers, psychological factors, smoking, host immune system and microbiota dysbiosis. However, the exact etiology of IBD is still not fully understood [79,80,81,82]. CD involves all parts of the GI tract from the mouth to the anus, whereas UC is confined to the colon. Activation of these cells in the intestinal mucosa contributes to elevated local levels of pro-inflammatory cytokines, such as tumor necrosis factor alpha (TNF-α), interleukin 1β (IL-1β), interferon-γ (IFN-γ), and interleukin-23 (IL-23), among which TNF-α attracts more attention due to its remarkable pro-inflammatory and proapoptotic effects [83]. Therefore, TNF-α blockers are central in IBD therapy. Besides cytokine production, the overproduction of different reactive oxygen species, including superoxide anion radicals, hydroxyl radicals, singlet oxygen, and triplet oxygen from activated leukocytes, overwhelm the tissue’s antioxidant defenses and could be another molecular event involved in IBD pathogenesis [84]. Oxidative stress is also suggested to have a crucial role in apoptosis. It has been exhibited that the concentration of antioxidants (vitamins, flavonoids and trace elements) in patients with CD and rat experimental models of UC is markedly lower [85]. Antioxidants, defined as substances that significantly delay or inhibit oxidation of an oxidizable substrate, can be beneficial in IBD therapy even when used at concentrations lower than the oxidized substrate [86]. More than 230 genes predisposing people to IBD have been discovered [87]. Many of these IBD susceptibility genetic polymorphisms are associated with host mucosal barrier function and are involved in host–microbiome interactions [88,89,90], thus supporting the hypothesis that alterations in the gut microbiome are essential for triggering chronic inflammation, and not merely a consequence [91,92].

In addition, nuclear factor-kappa B (NF-κB)-signaling significantly contributes to multiple host responses underlying IBD pathogenesis. The NF-κB family of transcription factors are regulators of inflammation and gut epithelial integrity, and activators of antigen-presenting cells and effector leukocytes [93]. Upon activation, NF-κB dimers translocate to the nucleus, where they modulate the transcription of several genes, including those involved in inflammatory and immune responses [94]. Several IBD genetic risk alleles, including nucleotide-binding oligomerization domain-containing protein 2 (NOD2), TNF-α-induced protein 3 (TNFAIP3/A20), and Toll-interacting protein (TOLLIP), promote gut pathogenesis, at least partly, through dysregulated NF-κB signaling [95]. Epithelial cells and macrophages isolated from the inflamed intestine of IBD patients show increased activation and nuclear localization of NF-κB-p65 [96]. Aryl hydrocarbon receptor (AHR)–NF-κB–CCAAT enhancer-binding protein beta (C/EBP-β)-signaling axis is found to operate in T cells and dendritic cells to promote intestinal inflammation [97].

Last but not least, the nuclear-related factor 2/ Kelch-like ECH associated protein 1 (Nrf2/Keap1) signaling pathway seems to regulate GI tract function, and therefore may moderate the course of IBD. According to Arisawa et al. [98], an Nrf2 gene polymorphism may be associated with the development of UC, while Myers et al. [99] supported that Nrf2 levels are found to be decreased in UC patients and those with active CD compared to healthy controls. On the other hand, higher Nrf2 levels are positively linked to antioxidative enzymes such as peroxiredoxin-1 and glutathione S-transferase A4, and negatively linked to pro-inflammatory IL-17a [100]. A study reported that persistent Nrf2 activation is the adaptation of colonic epithelial cells to oxidative stress during chronic inflammation of active IBD [101]. Stimulation of Nrf2 may be linked to the excessive mobilization of NF-kB during inflammatory reactions [68,69] and, in turn, may increase ROS production and enhance immediate early response-3 protein (IER3) expression [102,103]. The effect of Nrf2 on IBD development and progression should be further investigated. 

### 3.3. Therapeutic Options

5-Aminosalicylic acid, sulfasalazine, glucocorticoids, azathioprine, 6mercaptopurine, thioguanine, methotrexate, cyclosporine, infliximab, and tacrolimus are all used for IBD treatment. However, the long-term usage of these drugs is limited due to several side and adverse effects, such as diarrhea, abdominal pain, nausea, and vomiting (sulfasalazine), pancreatitis, hepatotoxicity, and hematologic problems (methotrexate, azathioprine), leucopenia (6-mercaptopurine), nephrotoxicity, cardiotoxicity, hypertension, gingival hyperplasia, and arteriolopathy (cyclosporine and tacrolimus) [104]. Moreover, about 30–50% of CD patients and 15–20% of UC patients are not sufficiently controlled with conventional anti-inflammatory treatment, and most of these treatments are inadequate [105]. Conventional anti-inflammatory agents serve as symptomatic therapeutic means, whereas concerns have been raised regarding the efficacy of novel biologic and small molecule-targeted therapeutics. Consequently, heterogenous recommendations have been provided, which usually do not include them [106]. Surgical intervention is required in complicated cases, or when pharmacological treatment is not successful. Regarding the cited problems, new therapeutic strategic approaches should be used for IBD therapy. Targeting of CD4+ T-cell cytokines, inhibition of leucocyte adhesion and gene therapy are amongst the newest treatments proposed for IBD; however, it should be mentioned that many patients (from 39% in Austria to 51% in Germany) turn to herbal therapy in addition to conventional treatments [107].

### 3.4. Natural Products

Various herbal products, such as Portuguese blueberries and dark purple rice extract, have been used for IBD treatment [108,109,110,111]. Many investigations have reported that plant derivatives, such as polyphenols, are involved in a wide range of biological activities, such as anti-inflammatory and antioxidative activities, as secondary plant metabolites [112]. Dietary fiber showed an anti-IBD effect through the modification of TNF-α, IL-2, and nitric oxide (NO). Natural supplementation has a central role in cytokine production regulation. 

## 4. Therapeutic Effects of C3G in IBD 

Nowadays, more and more patients with IBD prefer an alternative medical approach to their condition, and turn to plant-based drugs; thus, anthocyanin-rich foods are highly emphasized. ACNs and anthocyanidin glycosides have presented an important role in preventing and treating IBD. C3G has been recognized to have antioxidative, anti-inflammatory, cytoprotective, and antimicrobial effects that can be useful for IBD management (Table 1). In particular, C3G has been shown to act directly as an antioxidant and free radical scavenger [36,113,114]. Additionally, it has been found to regulate several detoxification enzyme pathways [115,116], modulate cytokine levels [117] and affect IFN pathways [39,117]. It also has an indirect effect via modulation of transcriptional factors, including NF-κB [114,118,119,120,121] and MAPK [122,123] activation and the Nrf2 pathway [116,119]. G3C can also improve intestinal microbiota composition [120,124].

### 4.1. C3G Inhibits NF-κB Pathway Activation

NF-κB is the main redox-sensitive nuclear transcriptional factor involved in the intestinal inflammation process [127], and plays a role in the regulation of several pro-inflammatory mediators, including interleukins (IL-6, IL-8, IL-12, IL-1β), TNF-α and IFN-γ [128]. Although NF-κB is involved in preserving intestinal epithelial cell homeostasis and regulating intestinal permeability [129], the chronic activation of NF-κB is typical of IBD and may play a critical role in the aggravation of inflammatory conditions in the intestinal epithelium [96,130]. Many drugs for IBD interfere with the activation of NF-κB [131,132]; thus, natural antioxidants and their compounds that modulate the NF-κB pathway, such as C3G, have great potential as efficient complementary approaches for IBD [133,134].

Numerous in vitro studies have demonstrated the association between C3G and the NF-κB pathway [36,114,118,119,121]. A study by Cremonini et al. [118] indicated that C3G was able to completely prevent TNFα-mediated increases in IKKα and p65 phosphorylation in Caco-2 cells. Protection of TNF-α-induced activation of NF-κB led to a 1,4-fold increase in the MLC kinase-mediated phosphorylation and activation of MLC. It was therefore suggested that inhibition of the NF-κB pathway was the primary mechanism of monolayer protection from TNFα-induced decreases in TEER and increases in FITC-dextran permeability. Likewise, Speciale et al. [119] claimed that C3G’s anti-inflammatory effects on TNF-α-induced Caco-2 cells were mediated via inhibition of the NF-κB pathway activated by TNF-α, and a reduction in IL-8 and IL-6 mRNA levels.

A study by Bashllari et al. [36] indicated a dose-dependent inhibition of the upregulation of palmitic acid (PA)-induced transcriptional activity of p65, IL-6, and IL-8 mRNA levels in PA-induced Caco-2 cells; thus, C3G helped in restoring gene expression levels to control values. Cytokines’ downregulation was also reported in cells not exposed to PA. During the same study, the anti-inflammatory effect of C3G was further evaluated, considering another NF-κB downstream target, COX-2. It is worth mentioning that the human gene encoding COX-2 has two binding sites for NF-κB, in correspondence with the promoter region. It is activated by TNF-a, IL-1, IFN-γ via the NF-κΒ and AP-1 pathway, and upregulated in IBD intestinal mucosa. According to Bashllari et al., C3G reduced COX-2 protein levels in a dose-dependent manner, confirming the protective effect of C3G against PA-induced Caco-2 cells.

Regarding inflammation, a recent study by Tan et al. [113] reported that C3G monomer and C3G-BP complexes have been found to downregulate iNOS and COX-2 protein expression in Caco-2 cells. The greatest anti-inflammatory effect has been observed in HHP-treated C3G-BP complexes. According to Ferrari et al. [114], a C3G dose of 20–40 μM administered to intestinal Caco-2 cells exposed to TNF-α has anti-inflammatory properties through inhibition of the NF-kB pathway. C3G reduces TNF-α levels, IIKKα/β phosphorylation/activation and IκB, the two upstream kinases regulating NF-kB. Furthermore, it inhibits the expression of Il-6 induced by TNF-α, and downregulates COX-2, PGE2, and TXB2 [114]. Noting that the endothelium is closely linked to the initiation and propagation of IBD pathology, while distinctive features of the intestinal endothelium contribute to these conditions [135], Ferrari et al. [121] investigated C3G’s modulatory effects on in vitro inflammatory crosstalk between intestinal epithelial and endothelial cells, using Caco-2 and HUVECs cells. 

IBD initiation and progression stages are well known to involve changes in mucosal immunity and gastrointestinal physiology. Endothelial cells adjust structurally and functionally to modulate vascular supply, immune cell emigration, and the tissue environment [136]. In active IBD, angiogenesis of the endothelium mediated by chemokines and cytokines might correlate with disease severity. The newly formed endothelium or inflamed vessels differ from normal vessels in the terms of production of and response to pro-inflammatory cytokines, adhesion molecules and growth factors [137]. As a result, barrier function, coagulant capacity and blood cell recruitment after injury might be altered in the newly formed endothelium. Thus, Ferrari et al. [121] concluded that selective inhibition of the NF-κB pathway in epithelial cells represents the main mechanism by which C3G exerts its protective effects. C3G has been reported to downregulate TNF-α-induced nuclear translocation of NF-κB, reduce TNF-α and IL-8 gene expression in Caco-2 cells, and subsequently downregulate endothelial cells’ activation, while decreasing E-selectin and VCAM-1 mRNA levels, leukocyte adhesion, and NF-κB levels in HUVECs [121].

Min et al. [122], using RAW 264.7 cells exposed to LPS, confirmed the downregulatory effects of C3G on TNF-α, L-1β, NO expression, LPS-induced PGE2, and NOS levels. Several IBD model studies have shown that C3G exhibits anti-inflammatory activity via NF-κB inhibition [122,124]. According to Tan et al. [124], high-pressure threatened (HHP)-C3G-BP complexes have more enhanced anti-colitic effects than C3G monomers in DSS-induced colitic UC mice; HHP treatment increases ACNs’ stability and availability in the body [138]. In the study, C3G administered orally at 200 mg/kg together with blueberry pectin was reported to be effective in alleviating inflammation by inhibiting NF-κB, as indicated in the significantly reduced levels of p65 expression [124]. In the study by Min et al. [122], in which oral administration of C3G was evaluated in BALB/c carrageenan-induced inflamed mice, C3G led to inhibition of NF-κB activation and pro-inflammatory mediation of COX-2 expression. Similarly, administration of C3G at a dose of 40 mg/kg for five days to antibiotic-associated diarrhea BALBc mice seemed to have anti-inflammatory effects through reducing the level of p65 phosphorylation and TNF-α, IL-6, and IL 12 levels, thus inhibiting the inflammatory facilitation of the NF-κB pathway [120].

Activation of the redox-sensitive signals IKK/NF-kB and increased expression of the PTP1B phosphatase regulated by NF-kB are cited in a study conducted by Daveri et al. [139] on mice fed with a high-fat diet and an AC-rich blend. Increased NF-kB p65 nuclear translocation of TNF-α challenged cells was additionally confirmed in in vitro studies using human umbilical vein endothelial cells [123]. A study by Fratantonio et al. [140] similarly found that C3G significantly inhibited the NF-κB pro-inflammatory pathway and adhesion molecules induced by PA; therefore, these effects have been attributed to the activation of Nrf2/EpRE pathway.

### 4.2. C3G Activates Nrf2 Pathway and Modulates Cytoprotective Enzymes Expression

Some bioactive components of food, including ACNs, exert indirect antioxidant activity by regulating the expression of antioxidant enzymes and cytoprotective proteins, such as NAD(P)H, superoxide dismutase, heme-oxygenase (HO-1), thioredoxin, quinone reductase-oxide 1 (NQO1), catalase, glutathione S-transferase and glutathione peroxidase, which are essential for cell protection [123,141]. Increased expression of these molecules could be modulated by Nrf2, a member of the NF-E2 family (transcription factors with basic leucine zipper domains). Nrf2 and its target genes mainly exert antioxidative effects or protective effects from chemical-induced cellular damage. Nrf2 is sequestered in the cytoplasm by Keap1; phase II enzyme inducers and prooxidants can induce its modification and disrupt the Nrf2–Keap1 complex, causing Nrf2 translocation into the nucleus, where it binds to the antioxidant responsive element (ARE), which is a cis-acting enhancer element that stimulates gene expression [142].

A study conducted by Speciale [119] indicates that C3G exerts an indirect antioxidant cell-adaptive response through the activation of the Nrf2/ Keap1 pathway in Caco-2 cells exposed or not to TNF-α. Pretreatment with 0.75–1.5μg C3G eq./mL induced overexpression of NQO1, which is a gene present in the ARE sequence in cells exposed to TNF-α or not. Another in vitro study conducted using Caco-2 cells and palmitate to induce a lipotoxic environment [36] illustrated that C3G has been able to increase the expression of NQO1. In addition, it seemed to activate the Nrf2 pathway. According to Bashllari et al. [36], C3G’s anti-inflammatory effects may be attributed to an antioxidant adaptive cell response mainly regulated by the Nrf2 pathway. On the other hand, given the inhibitory effect that the Nrf2/EpRE pathway may have on NF-κB transcription machinery [140], a hypothesis of the crosstalk between Nrf2 and NF-κB pathways that could modulate the transcription or function of target proteins has been supported. In a study by Ferrari et al. [114], Caco-2 cells were exposed to TNF-α and treated with 20–40 μM C3G for 24 h. C3G was able to increase Nrf2 translocation in a dose-dependent manner, and HO-1 and NQO-1 mRNA Levels both in TNF-α treated and unexposed cells. Therefore, Ferrari et al. [114] support that the upregulation of the Nrf2 pathway is involved in C3G’s protective effect on epithelial inflammation induced by TNF-α. 

The In vitro study by Serra et al” [116] indicates that C3G can induce the activation of Nrf2 in cytokine-stimulated HT-29 cells. In fact, the stimulatory effect of C3G alone has been shown to be significantly higher than that assigned to 5-ASA, particularly when considering differing concentrations (25 μM C3G vs. 500 μM 5-ASA). Notably, the combination of 5-ASA with C3G failed to reveal an additional/synergistic effect. Finally, C3G was found to be responsible for the increase in the HO-1 mRNA expression Moreover, it enhanced the GSH/GSSG ratio and slightly increased the mRNA expression of catalytic and modified subunits of GCL in cytokine-exposed cells. Nrf2/ARE activation by C3G was also confirmed in endothelial cells [123]. The results of the in vitro study conducted by Speciale et al. suggest that C3G activated the NRf2/ARE pathway at baseline and after TNF-α treatment of HUVECs, which in turn regulated several detoxification enzyme pathways such as HO-1. The study suggested the involvement of specific mitogen-activated protein kinases (MAPKs) (ERK1/2) in C3G’s induction of the Nrf2/ARE pathway. Finally, an inhibitor’s inactivation of ERK1/2 activity abolished the increase in Nrf2 nuclear accumulation induced by C3G [123]. 

### 4.3. C3G Modulates IFN Pathways

CD169 is expressed by some specific immune cells, especially dendritic cells (DCs) and macrophages. CD169+ macrophage subsets are mostly located in secondary lymphoid organs such as the subcapsular sinus. Medullary macrophages in lymph nodes (LN) highly express CD169, just as marginal metallophilic macrophages in the spleen do [143,144,145,146]. CD169+ macrophages encounter and engulf invading microbes at the entry sites of lymph or blood, acting as a gatekeeper within their special location at which antigens enter, and also act as activators of T and B cells to mount an immune response against pathogens [147,148]. Colonic CD169+ macrophages are mostly located in the lamina propria of the colon. After epithelial injuries, CD169+ macrophages produce CCL8 to initiate mucosal inflammation by recruiting inflammatory monocytes [143,149,150]. CD169-DTR mice with deleting or decreasing CD169+ macrophages did not display the typical clinical symptoms of colitis induced by DSS. Some studies have indicated that CD169+ macrophages play a crucial role in the development of colitis [151,152]. 

Xia et al. [117] evaluated the effects of orally administered C3G on DSS-induced colitic mice to conclude that C3G prevents the increase in CD169 cells. Furthermore, in vitro C3G administration could have directly inhibited macrophage activation and CD169+ cells’ numerical increase. In the colon and mLNs, C3G has been found to reduce pro-inflammatory cytokine expression, including IL-6, IL-1β, IL-18, IL-17, TNF-α, and IFN-γ, to reduce CD169+ macrophages, and to increase CCL22 expression; some anti-inflammatory cytokines were also increased, e.g., IL-10 and TGF-β. C3G administration resulted in a reduction in CD80 and CD86 expression and induction of Tregs, together with an increase in CCL22 expression levels in both the colon and mLNs. The activation of peritoneal macrophages has been reported to be inhibited by C3G in vivo, while the expression of CD80 and CD86 is decreased. Thus, C3G is reported to be able to reduce the number of peritoneal CD169+ macrophages and also to inhibit their activation. In the same study [117], C3G inhibited the CD169 expression induced by type I IFN. Thus, Xia et al. support the hypothesis of Lee et al., stating that NF-κB inhibition after administration of C3G [153] is a critical factor in the type I IFN pathway [154]. On the other hand, for the inhibition of the type II IFN pathway, a few studies have reported downregulation of IFN-γ caused by C3G [39,115,125,126]. The in vitro study of Triebel et al. [125] conducted using CM-stimulated T84 cells reported a downregulation of IP-10 (CXCL10) by C3G and a decrease in IFN-γ-induced protein levels, while reporting no significant downregulation of TNF-α and IL-8. 

### 4.4. C3G Reduces Reactive Species and Pro-Inflammatory Cytokines 

Reductions in pro-inflammatory cytokines after C3G administration have been confirmed by several studies. Such pro-inflammatory cytokines include IL-6 [36,117,119,120,121], IL-8 [36,113,115,119], IL-1β [36,116,117,122], TNF-α [117,119,120,124], IL-17, and IL-18 [117]. A cohort study by Liso et al. [126] on IBD patients receiving infliximab reported that supplementary administration of purple corn, which includes 0.5mg C3G equivalents/g DW, to their normal diet was associated with the downregulation of inflammatory biomarkers such as CRP, IFN-γ, TNF-α, IL-5, IL-9, IL-10, IL-12p70, and IL-17A, in CD but not UC patients. In addition, Serra et al. [115] claim that stimulation of a cytokine cocktail may be related to the suppression of an alternative cell signaling, one other than NF-kB. Administration of C3G to cytokine-stimulated HT-29 cells led to downregulation of NO, PGE2, IL-8, iNOS, COX-2, and STAT1 [115]. Similarly, according to Ferrari et al. [121], the downregulation of IL-6 induced by TNF-α, COX-2, PGE2, and TXB2, and the upregulation of GSH, HO-1, and NQO-1 mRNA levels induced by C3G underline C3G’s activity as a direct redox scavenger downregulating TNF-α. Many in vitro studies have revealed the reactive oxygen species (ROS)-mitigating potential of C3G [36,113,116]. C3G is claimed to induce a direct reduction in reactive species and an upregulation in several detoxification enzyme pathways, such as HO-1 [114,116], NQO-1 mRNA levels [36,114], GCLC, GCLM (glutamate cysteine ligase mRNA expression) [116] and GSH [114], and a change in the ratio of GSH/GSSG [116]. It has also been found that C3G and/or complexes with the monomer exert antiapoptotic properties [113,124,155]. Tan et al. reported increased protein levels of Bcl-2/Bax and the caspase-3/cleaved caspase-3 gene ratio, thus indicating the enhanced therapeutic effect of HHP-treated C3G on mice enteritis [113,124], the inhibition of the depolarization of mitochondria, and the reduction of the produced ROS, as demonstrated by the reduced mRNA expression of IL-1β, TNF-α, and IL-8. Increased expression of IL-10 and reduced iNOS, COX-2, Bcl-2, and cleaved caspase-3 levels were also observed [113]. However, a study by Xia et al. [117] found no significant effect of C3G administration in apoptosis. 

### 4.5. C3G Modulates Gut Microbiota 

Anthocyanins may modulate gut microbiota by inducing an increase in special gut bacteria [156] and increasing microbial abundances [157], thus mitigating dysbiosis. Anthocyanins have been claimed to increase the relative abundance of beneficial bacteria, such as *Bifidobacterium* and *Akkermansia*, which are believed to have anti-inflammatory effects [156,158]. In a previously referenced study by Wang et al. [120], C3G extracted from the Chinese bayberry has been positively correlated with an increase in richness and diversity of gut microbiota. Moreover, it was related to increased *Bacteroides* species, which are widely known for its beneficial effects and ability to reduce harmful bacteria, *Enterococcus* and *Clostridium sensu stricto 1*. C3G has been claimed to contribute to restoring the homeostasis of gut microbiota [120]. In detail, *Enterococcus* represented by *E. faecium* and *E. faecalis* is an opportunistic pathogen that helps the adhesion, colonization and invasion of host tissue, the modulation of host immunity, and the production of toxins and extracellular enzymes [159,160]. *Clostridium sensu stricto 1* has been found to proliferate in IBD, and is considered a potential biomarker of intestinal inflammation [161]. In addition, administration of C3G was positively correlated with the relative abundance of *Lachnoclostridium*, known for propionate and butyrate production, which contributes to restoring SCFAs concentration in the gut [162]. SCFAs are the ligands of two G protein-coupled receptors, Gpr43 and Gpr41, which participate in glycolysis and protein synthesis by modulating the level of some endocrine peptides. Moreover, they promote the proliferation, differentiation and apoptosis of intestinal epithelial cells. Finally, they protect the intestinal epithelial barrier [163,164]. *Parabacteroides* and *Blautia*, some beneficial bacteria, were also positively correlated with C3G administration. Tan et al. [124] indicated an increase in species diversity after C3G administration + HC treatment of DSS-induced colitic UC mice. C3G was found to induce a decrease in the relative abundance of *Firmicutes* and *Proteobacteria*, and an increase in *Bacteroidetes*, *Verrucomicrobia* and *Candidatus Saccharibacteria* of an HHP-treated C3G-BP group was the biggest change, resulting in a smaller *Firmicutes* to *Bacteroidetes (F/B)* ratio, which is related to the degree of inflammation in colitis [165]. In a study by Wu et al. that did not involve IBD directly [166], a mouse model of experimental non-alcoholic fatty liver disease was used. They demonstrated that the *Lonicera caerulea* L. berry, which is rich in C3G, was able to reduce inflammation due to the ratio change of *Firmicutes* to *Bacteroidetes*.

Aside from C3G, its metabolites have been investigated in a colitic environment. C3G’s metabolites, as components of the monofloral honey Prunella Vulgaris, have been found to exert protective properties in DDS-induced UC colitic mice by restoring the relative abundance of *Lactobacillus* [167]. More studies on C3G metabolites as components of food products have confirmed their anti-inflammatory effects through a reduction in the population of Bacteroides spp. In DSS-induced colitic rats [168], and through growth inhibition of *E. coli*, *S. aureus*, and *P. aeruginosa* [169], and therefore mitigation of the growth of pathogenic bacteria. Finally, phenolic compounds can be used as substrates by bacteria to produce energy [170,171] and to produce fermentable metabolites, which can exert bioactive functions similar to those of parent anthocyanins [172]. Thus, the gut microbiota play a role in the metabolism of anthocyanins and secondary phenolic metabolites after the removal of anthocyanins’ sugar moiety [173]. 

### 4.6. Clinical Aspects 

The cause of IBD is still not well understood. Current knowledge on IBD pathogenesis suggests genetically susceptible individuals develop intolerance to dysregulated gut microbiota, and chronic inflammation develops as a result of environmental triggers. Thus, there is limited evidence based on randomized controlled trials (RCTs) supporting that a substance alone or a specific diet pattern can prevent the disease. However, the scientific community should aim to find ways to manage IBD symptoms and improve gut health, mainly through gut inflammation alleviation, regulation of the immune responses involved in IBD, and improvement of gut microbiota composition. Thus, even though C3G appears very promising in treating and/or preventing IBD, further clinical studies should be conducted. Regarding administration, in vivo experiments in mice have mainly practiced oral administration and intraperitoneal C3G injections. Both routes of administration resulted in impressive findings regarding inflammatory responses, oxidative indices, and microbiota composition, but the latter was related to a greater reduction in immunomodulatory modules such as CCL22 and Tregs induction [117]. Concerning human studies, a cohort study investigated the effects of oral administration of C3G as a complex with a nutritional supplement, resulting in reduced inflammatory biomarkers in CD, but not UC patients [126]. Administrating C3G as a complex with other nutritional regimens is not an uncommon practice [124]. The limited stability, bioaccessibility and colonic delivery of the compound are considered to restrict the wider oral supplementary use of C3G alone against IBD. Nevertheless, loading of bioactive phytochemicals in a robust carrier system might be crucial to increase stability, solubility, intestinal absorption, and bioaccessibility, or to improve the bioactivity in body circulation through specific targeting. According to Shishir et al. [174], an efficient carrier of C3G, named nanofibersolome, has recently been developed to provide protection during its passage through the simulated digestion processes, thus being a very promising approach.

In addition, there are no registered interventional clinical trials investigating C3G’s effects on IBD patients. Regarding the wider category of anthocyanins, a randomized, double-blind, Phase iIa study was conducted by Rogler et al. [9], and evaluated the efficacy, safety and tolerability of an anthocyanin-rich extract (ACRE) in patients with UC (NCT04000139). During the trial, 3 g of anthocyanin-rich extract was administered daily in three doses of 2 × 500 mg, for 56 days. Another study regarding anthocyanins, expected to finish in 2024, is planning to evaluate the efficacy of Montmorency Tart Cherry Juice Supplementation on UC patients (NCT05486507). The administered dose reported in the majority of the in vivo studies in mice ranges from 24.2 to 96.8 g/kg BW or 1 ug daily [117]. However, during an aforementioned cohort study, 0.5mg C3G /g DW was administered to subjects as a complex with garlic acid [126]. Since clinical trials and cohort studies investigating the impact of C3G on IBD patients are limited, there are no clear recommendations on dosage. C3G levels can be evaluated via the plasma concentration of the compound, but also in urine and target tissues. 

To assess therapeutic outcomes after administrating C3G, validated scores could be used alongside a report of a series of clinical symptoms, laboratory tests, and endoscopic and histological assessments. The first validated score recommended is the Mayo score/disease activity index (DAI) for ulcerative colitis [175,176]; it evaluates stool frequency, rectal bleeding, mucosal appearance at endoscopy, and the physician’s rating of disease activity. The second one addressing CD patients is the Crohn’s Disease activity index (CDAI) [177], which scores on a scale of 0 to 100 and includes aspects such as abdominal pain, general wellbeing, complications, abdominal mass, anemia, and weight change. This composite instrument divides patients with CD according to their score into categories of asymptomatic remission, mild-to-moderate CD, moderate-to-severe CD, severe-to-fulminant disease, and clinically significant improvement in disease activity. Other scores that could be used in CD patients [178] are the Harvey–Bradshaw index, Crohn’s disease endoscopic index of severity (CDEIS) and the simple endoscopic score for Crohn’s disease (SES-CD). Last but not least, the capsule endoscopy Crohn’s disease activity index (CECDAI, or Niv score) [179] could be used to evaluate inflammation, the extent of CD, and the presence of strictures. Furthermore, therapeutic outcomes and clinical remission after administrating C3G could be assessed through evaluating parameters such as rectal bleeding, stool frequency, abdominal pain, endoscopic remission (colonic inflammation characterized by erythema, loss of normal vascular pattern, granularity, erosions, friability, bleeding [180] etc.), histological remission, physicians global assessment (PGA), fecal calprotectin, steroid dosage, and the SIBDQ (short inflammatory bowel disease questionnaire) [181]. Other parameters that could be evaluated include symptoms of urgency and fecal incontinence, weight loss and fever. As for laboratory tests, patients should be monitored for anemia, hypoalbuminemia, and elevated C-reactive protein (CRP). Elevated fecal calprotectin is a sensitive (but not specific) indicator of intestinal inflammation in IBD [182]. 

## 5. Conclusions 

**Figure 1:**
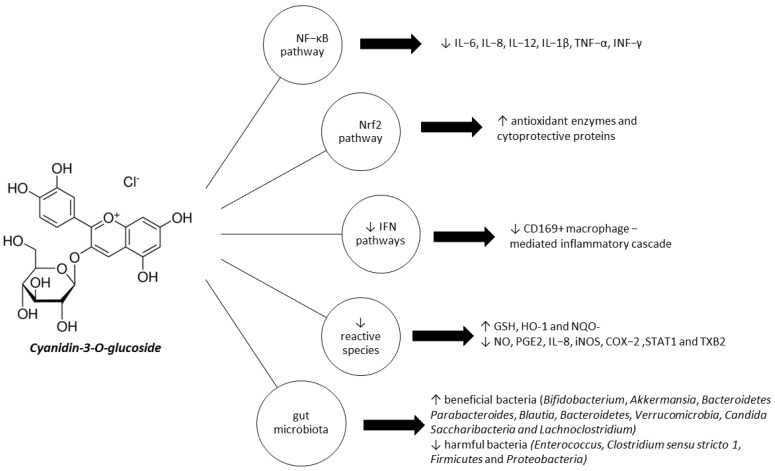
Therapeutic actions of C3G against IBD. NF-κB, nuclear factor-kappa B; Nrf2, nuclear factor erythroid 2–related factor 2; IFN, interferon; IL-6, interleukin 6; IL-8, interleukin 8; IL-12, interleukin 12; IL-1β, interleukin 1β; TNF-α, tumor necrosis factor alpha; IFN-γ, interferon-γ; GSH, glutathione; HO-1, heme oxygenase-1; NQO-, NAD(P)H Quinone Dehydrogenase 1; NO, nitric oxide; PGE2, prostaglandin 2; iNOS, inducible nitric oxide synthase; COX-2, cyclooxygenase 2; STAT1, signal transducer and activator of transcription 1; TXB2, thromboxane B2.

According to recent studies, mounting evidence suggests C3G might be a potential therapeutic target for preventing or controlling the progression of IBD, while alleviating symptoms (Figure 1) [113,114,117]. C3G is claimed to exert not only anti-inflammatory, antioxidative, and cytoprotective, but also immunomodulatory and anti-microbial effects. C3G acts both directly as a free radical scavenger, modulating detoxification enzymes and cytokine expression, and indirectly via downregulating the redox-dependent transcriptional factor NF-κB, upregulating the Nrf2 pathway, and improving the composition of the intestinal microbiota [116].

While current treatment options achieve sustained remission of IBD in most patients, upcoming treatment regimens involving different molecular pathways and modes of actions are further highlighting the need for personalized medicine [24]. In conclusion, C3G may represent a promising alleviating agent for IBD, while its metabolites may be important lead compounds for the development of new therapeutic tools against the disease. Although the potential effect of this compound has been reported across various in vitro and in vivo settings, more preclinical and clinical studies are required to validate its potency, determine the recommended therapeutic dose, frequency and route of administration, and to develop more efficient ways to exploit its several notable properties. 

## Figures and Tables

**Table 1:** The protective effects of C3G against IBD.

Year; Author	Study Type	Subjects (Animal/Cell Models/Individuals)	Dose	IBD Indicators	Related Molecular Mechanisms in Regulation of IBD
[125]	In vitro	CM stimulated T84 cells	25, 50, 100 μM for 4 h	NA	↓ IP-10 (CXCL10)
[115]	In vitro	Cytokine stimulated HT-29 cells	12.5 to 50 μM for 24 h	NA	↓ NO,↓ PGE2, ↓ IL-8, ↓ iNOS ↓ COX-2↓ STAT1
[116]	In vitro	Cytokine stimulated HT-29 cells	25 μM, for 1 h	↑ Nrf2 pathway, ↑ HO-1,↑ GCLC and GCLM ↑ GSH/GSSG	↓ Reactive species
[114]	In vitro	Caco-2 cells + TNF-α	20–40 μM for24 h	↑ Nrf2 pathway, ↑ GSH ↑ HO-1 and NQO-1 mRNA Levels	↓ TNF-α, ↓ IKKα/β phosphorylation/activation and IκBα, ↓ NF-κB pathway ↓ Il-6 induced by TNF-α, COX-2, PGE2 and TXB2
[118]	In vitro	Caco-2 cells	0.25, 0.5 and 1 μM for 24 h	↑ FITC-dextran permeability	↓ IKKα, ↓ p65 phosphorylation, ↓ MLC, ↓ TNFα, ↓ NF-kB pathway, ↓ TEER
[121]	In vitro	Caco-2-HUVECs	20 or 40 μM for 24 h	NA	↓ NF-κB pathway, ↓ TNF-α, ↓ IL-8 ↓ endothelial cells activation: ↓ E-selectin, ↓ VCAM-1 mRNA, ↓ leukocyte adhesion
[39]	In vivo and in vitro	BALB/c TNBS-induced colitic mice Caco-2 cell monolayer model +LPS	200 μL for 12 h before TNBS injection 24.2–96.8 g/kgBW daily for 3 days	NA	↓ MPO, ↓ TEER, ↓ LY flux values. ↓ NO, ↓ TNF-α, ↓ IL-1b, ↓ IL-6, ↓ IFN-γ ↓ histological damage
[117]	In vitro	(Cell culture: RAW 264.7 cells+ IFNα+ IFNβ, 24 h) Naïve mouse peritoneal macrophages, lymphocytes removed + 1 ug/mL LPS, 24 h	1 ug/mL for 24 h	NA	Direct inhibition of CD80 and CD86 Inhibition of CD169 Expression induced by Type I IFN ↓ IL-1β, IL-18, IL-6, IL-17, and TNF-α
[36]	In vitro	Caco-2 cells + 100 μM PA (basolateral side)	10 or 20 μM for 24 h	↑ Nrf2/EpRE pathway ↑ NQO-1	↓ NF-κB pathway ↓ IL-6 and IL-8 mRNA levels ↓ COX-2 ↓ ROS
[113]	In vitro	Caco-2 cells+ LPS ± HPP	C3G-BP complexes (100–100 μg/mL)	↑ IL-10	↓ depolarization of mitochondria, ↓ ROS ↓ IL-1β, TNF-α, and IL-8 ↓ iNOS, COX-2, Bcl-2 and cleaved caspase-3 levels Inhibition of apoptosis
[119]	In vitro	Caco-2 cells +TNF- α	0.18, 0.37, 0.75, 1.5 μg C3G eq./mL for 24 h (ACN-rich purified and standardized bilberry and blackcurrant extract (BBE))	Activation of Nrf2/ Keap1 pathway	Inhibition of NF-κB pathway activated by TNF-α ↓ IL-8 and ↓ IL-6 mRNA levels
[124]	In vivo	DSS-induced colitic UC mice + HHP treatment	HPP 200 mg/kg C3G+ blueberry pectin complex (Oral administration)	↑ protein levels of the ratio Bcl-2/Bax and caspase-3/cleaved caspase-3 genes ↑ *Bacteroidetes, Verrucomicrobia Candidatus Saccharibacteria.*	↓ mRNA expression of pro-inflammatory factors ↓ NF-κB P65, ↓ NF-κB pathway ↓ *Firmicutes, Proteobacteria*, ↓ *Firmicutes to Bacteroidetes (F/B)* ratio
[126]	Cohort study	47 IBD patients Administration of a purple corn supplement to IBD patients receiving infliximab	Purple corn supplement composed by 2 mg GAE/g DW (gallic acid equivalents per g of dry weight) and total anthocyanin content of 0.5 mg cyanidin 3-glucoside (C3G)	NA	CD group only, not UC: ↓ CRP, ↓ IFN-γ ↓ TNF-α, IL-5, IL-9, IL-10, IL-12p70, and IL-17A

PA: palmitic acid, HHP: high hydrostatic pressure treatment, BP: blueberry pectin, HUVECs: human umbilical vein endothelial cells, TEER: transepithelial electrical resistance, LY flux: Lucifer yellow flux, TNF-α: tumor necrosis factor-a, IL-6: interleukin-6, IL1b: interleukin-1b, IFN-γ: interferon-γ, PGE2: prostaglandin E2, iNOS: nitric oxide synthase, COX-2: cyclooxygenase-2, HO-1: hemoxygenase-1, MLC: phosphorylation of myosin light chain. GCLM: glutamate cysteine ligase mRNA; ↑, increase; ↓, decrease.

## Data Availability

Not applicable.

## Abbreviations

ACN, anthocyanidins; AhR, aryl hydrocarbon receptor; ARE, antioxidant response element; Bcl-2, B-cell lymphoma-2; CD, Crohn’s disease; C/EBP-β, CCAAT enhancer binding protein beta; CM, cytokine mixture; COMT, catechol-O-methyltransferase; COX-2, cyclooxygenase 2; Cy, cyanidin; CVD, cardiovascular disease; C3G, cyanidin-3-O-glucoside; DC, dendritic cells; Dp, delphinidin; DSS, dextran sodium sulfate; DW, dry weight; EGFR, epidermal growth factor receptor; EHC, enterohepatic cycle; ERK, extracellular signal-regulated kinases; FA, ferulic acid; GCLC, glutamate cysteine ligase catalytic subunit; GCLM, glutamate cysteine ligase regulatory subunit; GI, gastrointestinal; GPCRs, G protein-coupled receptors; GSH, glutathione; GSSG, glutathione disulfide; HDL, high-density lipoprotein; HHP, high hydrostatic pressure; HO-1, heme oxygenase-1; HUVECs, human umbilical endothelial cells; IBD, inflammatory bowel disease; ICAM-1, intercellular adhesion molecule1; IFN-γ, interferon gamma; IL-1β, interleukin 1β; IL-6, interleukin 6; IL-17, interleukin 17; IL-8, interleukin 8; IKK, inhibitor of nuclear factor kappa kinase; iNOS, nitric oxide synthetase; IP-10, inducible protein-10; IFN-γ, interferon-γ; KEAP1, Kelch-like ECH associated protein; LDL, low-density lipoprotein; LN, lymph node; LPH, lactase-phlorizin hydrolase; LPS, lipopolysaccharide; MAPK, mitogen-activated protein kinase; MCP-1, monocyte chemoattractant protein-1; MLC, myosin light chain; Mv, malvidin; NCCDs, non-communicable chronic diseases; NF-κB, nuclear factor-kappa B; NO, nitric oxide; NOD2, nucleotide-binding oligomerization domain-containing protein 2; NRF2, nuclear factor erythroid 2–related factor 2; OSCC, oral squamous cell carcinoma; PA, palmitic acid; PCA, protocatechuic acid; PC, phenolic compounds; PGA, phloroglucinaldehyde; Pg, pelargodin; PGE2, prostaglandin 2; Pn, peonidin; Pt, petunidin; PST, phenyl sulfotransferases; PTP1B, protein tyrosine phosphatase 1B; PGE2, prostaglandin E₂; ROS, reactive oxygen species; RSC, radical scavenging capacity; SCFAs, short-chain fatty acids; STAT1, signal transducer and activator of transcription 1; sVCAM-1, soluble vascular cell adhesion molecule-1; TEER, transepithelial electrical resistance; Th1, T helper cell 1; Th17 T helper cell 17; TNF-α, tumor necrosis factor alpha; TNFAIP3/A20, TNF-α-induced protein 3; TOLLIP, Toll interacting protein; TXB2, thromboxane B2; UC, ulcerative colitis; UGT, uridine 51 diphosphate glucuronosyltransferases; VCAM-1, vascular cell adhesion molecule 1.
